# Foods of the Future: Challenges, Opportunities, Trends, and Expectations

**DOI:** 10.3390/foods13172663

**Published:** 2024-08-23

**Authors:** Songül Çakmakçı, Bilgehan Polatoğlu, Ramazan Çakmakçı

**Affiliations:** 1 Department of Food Engineering, Faculty of Agriculture, Atatürk University, 25240 Erzurum, Türkiye; 2 Department of Food Technology, Technical Sciences Vocational School, Atatürk University, 25240 Erzurum, Türkiye; bilgehanpolatoglu@atauni.edu.tr; 3 Department of Nanoscience and Nanoengineering, Graduate School of Natural and Applied Sciences, Atatürk University, 25240 Erzurum, Türkiye; 4 Department of Field Crops, Faculty of Agriculture, Çanakkale Onsekiz Mart University, 17100 Çanakkale, Türkiye; rcakmakci@comu.edu.tr

**Keywords:** future foods, sustainability, food security, new food sources, alternative proteins, novel food, synthetic biology, safety, functional foods, nanotechnology, 3D food printing

## Abstract

Creating propositions for the near and distant future requires a design to catch the tide of the times and move with or against trends. In addition, appropriate, adaptable, flexible, and transformational projects are needed in light of changes in science, technology, social, economic, political, and demographic fields over time. Humanity is facing a period in which science and developing technologies will be even more important in solving food safety, health, and environmental problems. Adapting to and mitigating climate change; reducing pollution, waste, and biodiversity loss; and feeding a growing global population with safe food are key challenges facing the agri-food industry and the food supply chain, requiring systemic transformation in agricultural systems and sustainable future agri-food. The aim of this review is to compile scientific evidence and data, define, and create strategies for the future in terms of food security, safety, and sufficiency; future sustainable foods and alternative protein sources; factors affecting food and nutrition security and agriculture; and promising food systems such as functional foods, novel foods, synthetic biology, and 3D food printing. In this review, the safety, conservation, nutritional, sensory, welfare, and potential challenges and limitations of food systems and the opportunities to overcome them on the basis of new approaches, innovative interpretations, future possibilities, and technologies are discussed. Additionally, this review also offers suggestions for future research and food trends in light of future perspectives. This article focuses on future sustainable foods, alternative protein sources, and novel efficient food systems, highlights scientific and technological advances and new research directions, and provides a significant perspective on sustainability.

## 1. Introduction

With global population growth, the urgent demand for food and water, which constitute the basis of life, intensifies [1,2]. While the world’s traditional biological resources are being depleted, the need for healthy and sustainable food resources is increasing. Although food security has improved to a certain extent in recent years, access and security to food, the importance of which has begun to be realized by humanity as it becomes more complicated during and after disasters such as the COVID-19 epidemic and earthquakes [3], is a serious problem faced by many countries and regions [4].

Food systems are the largest consumers of freshwater and are responsible for the majority of greenhouse gas emissions and biological loss caused by pollution due to fertilizers and pesticides [5], as well as being a major contributor to climate and land use change, the depletion of freshwater resources, and the pollution of aquatic and terrestrial ecosystems due to excessive nitrogen and phosphorus inputs [6,7]. Rapid urban expansion and industrial development cause resource scarcity. While the challenge of widespread hunger and malnutrition continues in many parts of the world, FAO [8] emphasized that the current rate of progress will not be sufficient to eliminate hunger by 2030 or even by 2050. In addition to the climate crisis, the pressure that consumption and production systems place on natural resources endangers food systems [8]. An increase in extreme climatic events slows or even negatively affects the positive effect of technological progress on yield [9,10]. Climate change alters the water cycle process, affects the irrigation water supply, and affects food security by increasing the frequency and intensity of extreme climate events [11]. While rainfall and groundwater are gradually decreasing, the duration and frequency of rainfall are increasing [12], and effective intervention is needed for the sustainable and effective use of land and water [13].

Along with people’s search for healthy and enjoyable nutrition, population growth, changing demographics, climate change, the depletion of natural resources, inequitable food distribution, decreases in the quality and adequacy of food, food waste, economic and ecological disruption, water shortages, land degradation, vulnerability to natural disasters, insecurity, environmental and public health, and resource constraints pose great challenges in terms of a sustainable and healthy food supply in the future. Soil, water, and environmental pollution, water loss, nutrient loss, soil erosion, air pollution, biodiversity loss, and climate change are depleting the planet’s food production systems [6,14,15]. Their negative environmental impacts and lack of resistance to threats to food security [16], climate change [17], and water security [18] indicate that current food production systems are not fully fit for purpose. As has been observed in some cases in recent years, even advanced technologies and control systems cannot guarantee food quality and safety.

In today’s world, where natural resources are polluted, mismanaged, and seriously depleted, developing a sustainable food system that will feed the increasing population while protecting ecosystems and natural resources continues to be a challenging task. Providing sufficient, environmentally sustainable, nutritious, safe, and accessible food for everyone is at the top of sustainable goals. It is important to raise awareness about sustainability in the food industry. Food in the future aims to address the global food supply, food security, nutrition, and health problems by producing healthier, safer, more nutritious, and more delicious food to guarantee human survival [19,20]. However, new and efficient sustainable food systems are needed because the current long supply chain-based food system cannot feed the global population and creates negative ecological, environmental, logistic, and nutritional pressures [21]. Agricultural systems are not compatible with global targets for food and nutrition, climate, environment, and livelihood security [22], and may even become victims of the environmental degradation they cause. Therefore, major transformation is needed to ensure nutrition and food security, as well as to meet climate, diversity, and health goals [23]. In the future, food systems will need major changes to increase food production by using fewer resources and reducing food waste. With food safety and sustainable food production, nanoparticles, nanoemulsions, conjugates, microorganisms, functional materials, modified biopolymers, and genes are becoming increasingly important issues in the field of food science [24]. In the food industry, the emergence and development of new foods and new food industries, the evolution and increased use of functional foods, and the use of nanotechnology have been reviewed as increasing trends. However, the precautionary principle should not be ignored, as the food opportunities provided by innovation processes and technologies may have unknown effects on health [25]. In this review, future sustainable foods and alternative protein sources, factors affecting food and nutrition security, and promising food systems are evaluated, and suggestions for future research and food trends are offered.

## 2. Food Quality, Security, Safety and Sufficiency

Food security strategies focus not only on the quantity of food but also on food quality, which consists of nutritional values such as vitamins, mineral elements, and proteins, as well as sensory, mechanical, and functional properties [26]. It may also be that subjective quality, which expresses how the consumer perceives quality attributes, gradually replaces objective quality, which expresses the physical product characteristics that should be desired by consumers. Once safer and more stable products are obtained, the nutritional and sensory aspects of food become the goals of process design. Past, present, and future challenges in food processing are reported to be related to safety, conservation, nutritional, sensorial, well-being, and environmental issues and emerge as sea waves across time [27]. Although the primary purpose of food is nutrition, people make food choices on the basis not only of taste and nutritional value and the presentation of foods but also of cultural, religious, historical, economic or social status, and environmental factors [28]. Food technology, which initially started with the challenges of guaranteeing the integrity, stability, and safety of food, has gradually focused on the development of food products with enhanced flavor and, subsequently, health and well-being [27,29]. Although safe, stable, and nutritious products are obtained with minimum cost and nutrient degradation, sensorial aspects such as appearance, odor, flavor, taste, and texture must also be considered.

Food safety management is a multifaceted concept covering the environment, food, economics, and agricultural science [30]. Food security, which includes the availability and accessibility of sufficient, safe, culturally acceptable, nutritionally adequate, healthy, and nutritious food to meet nutritional needs and lead a healthy life [31,32], focuses not only on quantity but also on the quality of food [26]. In addition to food supply, food security is related to regional accessibility, food supply, utilization, residents’ purchasing power, food quality, and political and socioeconomic stability [4]. Food security includes ensuring that people have access to sufficient food for a productive life, as well as their evolving demands for fresh, authentic, convenient, and delicious products [24].

For a healthy life, sufficient, safe, nutritious food must be physically and economically accessible to meet the needs and preferences of all individuals. Although the essence of food security is access to safe and nutritious food, studies and agricultural systems still seem to focus mostly on food sufficiency. The availability, access, utilization, and stability of the food supply over time [33], as well as ensuring the safety, nutritional quality, diversity, and balance of food, are essential elements of food security. Food security and healthy nutrition also require functioning health systems, education systems, water and sanitation, transportation, energy, etc., and even dietary diversity, which requires agricultural diversity and biodiversity.

Specific recommendations for ensuring food security include agroecology, sustainability, and ecological intensification; increasing the resilience of ecosystems; maintaining soil health and reducing producers’ vulnerability to economic risks [3]; transforming food systems [16]; transitioning to a circular and resource-efficient economy and implementing the 3R principles of the circular economy—reduce, reuse and recycle [34,35]; reducing waste and pollution from agricultural contaminants [36]; providing a variety of fertilizers, crop varieties, and irrigation methods [37]; increasing the use efficiency of nitrogen, phosphorus and potassium fertilizers [38]; increasing the photosynthetic optimum and water and nitrogen use efficiency in cultivated plants [39]; developing appropriate agricultural scale operations [40]; supporting and encouraging small-scale farmers to integrate organically into modern agriculture [41]; adopting measures to increase the purchasing power of households in rural areas [42]; altering diets and eating more plant-source foods [43]; optimizing the composition of residents’ diets; reducing food waste; and adjusting the composition of grain consumption [44,45]. The importance of newly emerging techniques and materials for ensuring food quality and safety is increasing [24]. Improving food security would have positive impacts on food access and utilization.

The first challenge to be solved from the beginning of food processes is safety. Although ancient, it still exists and will remain relevant as a future challenge as new products and technologies are developed [27]. Current production systems fail to solve the problem of healthy nutrition without compromising the preservation of regional balance and ecosystem health, creating environmental damage and social injustices [3,46]. After safety, protection is a challenge because it guarantees the microbial, chemical, physical, and biochemical stability of safe products [27]. Food demand should be addressed sustainably by minimizing environmental impacts and maximizing social opportunities, but this is not always considered possible. While the unequal distribution of production and income increases food access problems, changes in income and prices negatively affect the balance between nutrition and diet [47]. Although the effects of resource scarcity and climate change on food security are known, while food sufficiency is prioritized, the future of food safety and nutritional quality are not considered [48]. The pressure to increase yields has encouraged intensive production systems, the food industry has concentrated on larger organizations, high-yield sensitive species, and varieties have caused biodiversity and environmental effects, and accessible high-quality fertilizer and other resources have begun to decline. Regardless, sustainable food security, which is an integral component and prerequisite for system resilience, food security, and nutrition, remains the ultimate goal, and smart choices and strategies are needed to achieve these multiple goals.

## 3. Alternative Protein Sources for Human Nutrition

### 3.1. Trends for Plant-Based Proteins

**Figure 1:**
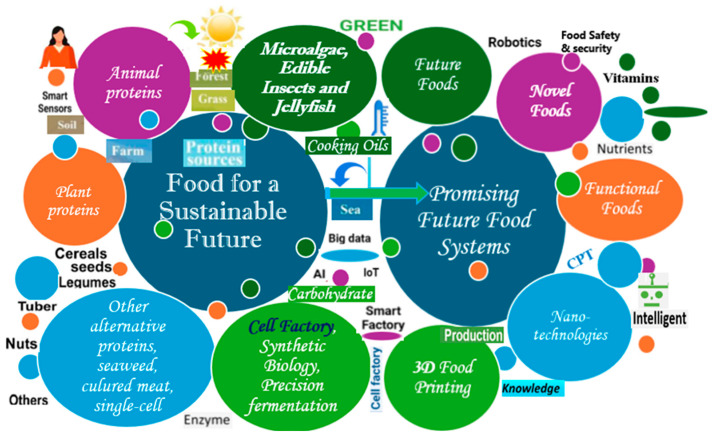
Main sources of protein and promising future food systems.

Although meat and dairy products are important sources of protein in human nutrition, in terms of current market developments and sustainability, animal proteins need to be partially replaced by plant-based proteins such as cereals, pseudocereals, oilseed, peas, beans, legumes, grass, green leaves, seeds and nuts, potatoes, mushrooms, seaweed, algae, etc. [49,50,51]. The main sources of protein and promising future food systems are shown in Figure 1. While pollution, greenhouse gas emissions, and environmental problems due to meat production reduce the consumption of animal-based food [52], it has been observed that the COVID-19 epidemic has contributed to the conversion of human diets, especially plant-based diets [53]. Plant-based proteins have a long history, lower production costs, are easy to access, and are more environmentally sustainable [54], and within limited natural resources, plant-based foods and proteins are a growing trend [50]. The Mediterranean diet, a predominantly plant-based nutritional model, has been recommended to reduce greenhouse gas emissions and water footprints and promote a sustainable lifestyle [55]. Food proteins obtained from animals are better digested than those obtained from plant sources [56], meat is one of the most important sources of dietary protein, and increasing populations and incomes also increase the demand for meat [57]. From the perspective of human health, environmental, and natural resources, a food system that moves toward fewer animal-based foods and more plant-based foods, such as minimally processed whole grains, legumes, vegetables, nuts, and fruits, is sustainable and beneficial [58,59,60]. Although there has been a consumer shift toward animal protein substitutes and plant-based dietary patterns due to health and environmental concerns in food systems [61,62,63], the tendency to reduce the consumption of animal products, which are ingrained in meat-rich Western culture, appears to be relatively low [64].

Increasing the adoption of plant-based diets is predicted to significantly reduce nutrition-related health problems, as well as agricultural and food-related greenhouse gas emissions, environmental impacts, and the demand for agricultural land, water, and fertilizer [65,66,67,68]. In addition to legumes used as food substitutes, grains such as wheat, rice, and oats and green leaves such as sugar beets are suitable sources. In fact, while wheat can be used with soy in meat substitutes, oats can be used as an alternative for dairy products because of their positive properties, such as supply, nutritional content, taste, and color, as well as an ingredient in other dairy substitutes, such as yogurt, cream, and desserts [51]. Although there has been a shift toward other protein sources, such as peas and chickpeas, soy is the primary source of plant-based proteins and an important alternative to meat and dairy products [51,54,69]. Legume proteins are an environmentally sustainable alternative to animal proteins [70].

Legume proteins such as soy, peas, chickpea, faba bean, kidney beans, and mung beans; cereal proteins such as wheat, corn, rice, sorghum and oats; and oilseed proteins such as peanut, flaxseeds, sesame, and sunflower are used as protein supplements [69,71]. In fact, the leaves of many plants, such as cowpea, sugar beet, alfalfa, and berseem, can be used as plant-based protein sources [51]. To reduce the consumption of animal protein, whose environmental impacts are concerning, alternative and sustainable protein sources with promising nutritional and environmental performance, such as algae, cyanobacteria, single-cell proteins, seaweed, fungi, mycoproteins, insects, jellyfish, cultured meat, and synthetic proteins, should be considered, as well as rich plant-based protein sources with low environmental impact [21,43,50,51].

Thousands of occasionally used and currently unused plant species may be used as food in the future [72], and discovered and undiscovered jellyfish, seaweeds, and aquatic animal species, which can be food sources without the need for soil, water, and fertilizer, are also potential foods for the future [73,74,75,76]. Brown, red, and green plant-like algae and marine biological resources are important resources that contribute to global food security and are used in food, feed, pharmaceutical, and biotechnological applications because of their high protein, vitamin, mineral, and bioactive compound contents and sustainability [74,75]. Plant-based foods constitute the largest portion of alternative proteins. In the future, byproducts of agricultural industries, such as rapeseed and sunflower seed meal, can be processed and used as protein sources to increase the profitability of food systems, while alternative cellular agricultural proteins produced from animal, plant, and microbial resources will become important [70]. The application of ultrasound to alternatives, which is a natural emulsion called plant-based milk, is a nondairy product containing peanuts, almonds, soy, and coconut; however, although similar to dairy milk, it has advantages such as physical stability, improved fermentation, and reduced pathogens [77]. Emerging food trends have significant potential for the development of sustainable alternatives to replace animal-based products [58], but there is a need for further research and evaluation of the impact of plant-based alternative foods on health, the environment, and nutritional quality [78].

### 3.2. Cellular Agriculture Proteins

Alternative proteins are important for future food security and for sustainable food production. While creating new types of foods and food ingredients may be possible through microbial fermentation, as a result of advances in synthetic biology techniques, stem cell biology, and tissue engineering, animal tissues can be produced in bioreactors using stem cells through the biotechnological production of alternative proteins such as cultured meat, although currently on a small scale [79,80]. Cellular agriculture is an emerging field for the production of different products and is promising for the production of cultured meat through tissue engineering techniques [81]. Although plant-based meat and laboratory-grown meat have been used as alternatives to conventional meat, their nutritional, economic, health, resource, and environmental impacts require extensive research [79,82]. Indeed, while leather, fish, egg, dairy, and seafood proteins have been successfully produced through cellular farming techniques, which are being promoted as a forward-looking new solution, cultured meat production is still at the research level [83]. In recent years, a development trend has been reported in the production of artificial meat, vegetable protein meat, and cell-cultured meat, which are thought to have advantages in terms of nutrition, health, safety, and environmental protection [20].

Although it has many technical difficulties [84], strategies are also being developed that reduce dependence on land and water requirements and natural resources and facilitate the production of cell-cultured meat, which is a healthy, safe, and sustainable alternative to real meat products in terms of nutritional value, taste, and aroma [85,86,87]. The production of animal tissue or cultured meat in bioreactors using tissue and stem cell culture in synthetic culture media [83] and precision fermentation, which programs microorganisms to produce specific products under certain conditions to produce cultured meat [86], has the potential to offer significant opportunities as innovative technologies. However, plant-based proteins still have the greatest potential to be used as a meat substitute, but the market potential may be low because the mass production of laboratory-grown meat, which is still in its infancy and experimental stage, is energy-intensive and not economically feasible, and has problems with consumer acceptance [88,89,90].

### 3.3. Microalgae, Edible Insects, and Jellyfish

Since meeting the increasing protein demand with only meat and dairy products is unsustainable in terms of land and emissions, plant-based foods and proteins, algae, cultured or in vitro meat, and edible insects have attracted attention as alternative protein sources. The closed environmental conditions enabled by food technology and food growing systems show that many alternative and risk-reducing foods can be grown on a large scale in controlled environments [91].

Algae: The use of algae, which are rich in proteins, minerals, vitamins, antioxidants, phytonutrients, and fatty acids, is increasing due to their functional benefits [92,93]. In fact, algae, which contain high amounts of protein and are also rich in essential amino acids, unsaturated fatty acids, and vitamins, can be added as functional ingredients to meat and meat-based products for healthy food production [94]. The production of microalgae, as an important source of sustainable and protein-rich foods, has been proposed to optimize their large-scale production in the future because of their potential to improve food safety and reduce resource and environmental problems [95]. The addition of algae to foods not only results in healthier foods but also extends their shelf-life [83]. The photosynthetic efficiency of microalgae could increase at optimized wavelengths in closed photobioreactors [96]. It requires significantly fewer resources and is more sustainable than livestock production [70].

Insects: Although there are some negative feelings about the consumption of insects as food, they are suitable for most human nutrition, and their use will become widespread because they require little space, land, water, and feed to grow [97]; have low greenhouse gas emissions and the use of nonrenewable resources [98]; have a significantly lower ecological impact than traditional livestock [99]; are rich in proteins, amino acids, fats, minerals, vitamins, and other nutrients [100,101]; and are sustainable and safe [70]. Greenhouse gas emissions, land, water, and energy use for insect production are generally lower than other animal and plant protein sources [98,102]. Insects, which constitute the largest biological group in the world because of their short life cycles, high abundance and reproduction rates, and low nutritional characteristics, are rich in various bioactive compounds and are considered a new potential resource for overcoming food crises and meeting nutritional needs [103,104]. However, ensuring safe conditions, including microbial safety, chemical contamination, and allergenicity concerns, requires further research, farming, processing, enzymatic hydrolysis, product evaluation, and cooking techniques [99].

From a nutritional perspective, insects, seaweed, and jellyfish are three good alternative protein sources, but optimal processing technologies and specific strategies for insect proteins are needed to promote their consumption, functionality, and sustainability [57,105]. The low environmental impact and high nutritional value of insect protein make edible insects a potential food for the future and a sustainable solution to food demand, but robust and high-throughput analytical methods must be developed to ensure authenticity, traceability, and safety against the risk of misidentification and counterfeiting [101,102]. Despite their ability to improve the nutritional profile of foods, low consumer acceptance and the lack of clear legislation for regulation are significant obstacles to their use as protein sources [99]. In the future, innovative insect-based products, as well as new regulations to take full advantage of the insect industry, are needed in terms of food safety and reliability.

Jellyfish: Although jellyfish are underused outside of Asia and scarce information is disseminated about their potential role, edible jellyfish is an environmentally sustainable alternative protein source that contains lipids, carbohydrates, vitamins, minerals, and collagen; is safe to use; and has certain organoleptic properties [68,106]. Although food safety raises concerns about compliance with appropriate legislation, it is a suitable alternative food source for human consumption because of its low energy, high protein, and very low cholesterol contents, with good safety [76,107].

## 4. Development Trends in the Promotion of Future Food Systems

### 4.1. Functional Foods

Overall, functional food has been defined as designed or modified products that have a more advanced role than just nutrient supply and gastronomic pleasure, that go beyond basic nutritional values, and that offer potential benefits in the prevention and management of disease [56,108]. These foods are fortified, enriched, or improved with a modified food or food ingredient that provides health benefits or reduces the risk of disease beyond basic nutritional functions. There is interest in functional foods such as fortified foods improved with nutrients and nutraceuticals, superfoods containing high amounts of nutrients and bioactive phytochemicals, and excipient foods that can increase the 3R ability of bioactive components in foods daily. Whether natural or industrially produced, functional and fortified foods contain ingredients and nutrients that are beneficial to nutrition and health [109,110], but the functionality of these compounds and the sensory properties of the product must be preserved [111]. Future large-scale food fortification with commonly and regularly consumed foods could improve the health and well-being of many people. In fact, economic growth and rising incomes increase the demand for foods with relatively high calorie and protein contents [112]. Owing to the bioavailability issues of traditional food fortification, such as the direct addition of nutrients to foods, the development of microencapsulation, stabilization, and fortification technologies has accelerated [113]. The increasing demand for functional foods and the disadvantages of traditional methods have accelerated efforts to develop new processing technologies aimed at preserving the functionality of bioactive compounds and the qualitative properties of foods. Nonthermal technologies are a reliable, efficient, and fast way to preserve the bioavailability of food components, such as the bioaccessibility of carotenoids, improve their functional and technological properties, and increase their recovery efficiency from agricultural products [56].

Innovative technologies have the potential to increase food production and sustainability, as well as improve food quality. Although nutrients and the bioavailability of nutrients are the main features of all types of food formulations, the structural properties and stability of soft solid products such as yogurt and consumer preferences, such as the sensory aspects of the food, texture, mouthfeel, color, and taste, are also important [56]. However, fortified foods can contain vitamins, minerals, antioxidants, probiotics, fatty acids, protein-like nutrients, and nutraceuticals such as carotene, polyphenols, phytosterols, and nanoparticles, which play a role in improving health and well-being, whereas nanoparticles can be used to encapsulate and protect nutrients and nutraceuticals [70,108]. Although dairy products are the most popular means of delivering probiotics to humans [114], in the future, it will become common to develop technologies that will improve the properties of new-generation probiotics as a part of existing probiotics [109].

The emerging market perspective leads food companies to develop many new functional foods, some of which fail and are withdrawn from the market because they are driven by technical feasibility rather than consumer acceptance. However, functional foods such as dairy products, meat, bakery, and beverages, which are developed by considering consumer awareness, preferences, attitudes, perceptions, high income, and high education and purchasing intentions, are becoming increasingly common. Among the functional foods that provide health, and physical and mental well-being beyond basic nutrition, the most common are probiotics and prebiotics.

The use of foods as probiotic carriers and the importance of the gut microbiota for health and well-being have increased the demand for probiotic foods [115,116]. Discovering and characterizing new microorganisms with multiple health-promoting properties, adapting them to food formulations, determining the probiotic potential of suitable unstudied fermented foods, and developing new dairy and nondairy probiotic foods should be explored [117].

### 4.2. Novel Foods

While artificial meat, milk, and eggs are developing as foods of the future [54], production technologies such as protein, fermentation, enzyme, cell and genetic engineering, and molecular food are the driving forces [118]. Although the applications of digital and technological innovations are limited, green technologies provide innovative solutions for the transformation of food systems [118], and interdisciplinary innovation continues to advance the global food industry toward total nutrition, high technology, and intelligence [119,120]. However, the opportunities offered by new technologies may lead to negative consequences if they are not developed with sustainability in mind. New technologies, products, and ingredients can contribute to keeping systems competitive and sustainable when resources are limited but can also create new food safety risks [48]. Fast technology can make effective and timely risk assessment difficult and may even increase risks from retail sales and food adulteration.

In addition to being rich in plant-based protein sources [49], foods produced from algae, fungi, bacteria, or photovoltaic-assisted microbial biomass, defined as microbial proteins or single-cell proteins, have been reported to be promising approaches that can contribute to food safety [121,122]. Microorganisms can be used in the production of some proteins and high-value functional ingredients, and microbial fermentation can be used in the production of milk proteins such as caseins [123]. In the future, innovative technologies, sustainable agriculture, nutritional changes, and the use of microorganisms such as fungi, bacteria, yeast, and microalgae to produce carbohydrates, proteins, and fats will become widespread [7]. With the food architecture approach, delicious, useful, healthy, and sustainable next-generation foods that look, feel, and taste like animal foods and are fortified with vitamins and minerals are created.

These novel foods and plant-based foods, including those obtained from forage fish, bivalves, mollusks, and insects, have become the focus of great interest in human nutrition worldwide for reasons such as environmental sustainability, high nutritional and production value, lower water and space requirements, greenhouse gas emissions and environmental footprints [44,124]. In fact, animal foods, with the exception of eggs, require more arable land than do plant foods, except for vegetable oil [125].

Driven by new technologies and innovations, an increasing number of plant-based alternative foods are being introduced to the market. In addition to being supplemented or extracted, processes can be slowed, accelerated, stopped, or renewed when necessary when foods are produced in the future [91]. Innovations in the food industry increase the number of new foods entering the market but create a climate of insecurity and avoidance for consumers [47]. However, the consumption of ultra-processed foods, which are encouraged by inappropriate eating contexts and socioeconomic, psychological, and lifestyle changes, can be a cause for concern owing to their generally low nutritional quality and poor nutritional profile [126]. However, it is clear that young people living in city centers are increasingly moving away from traditional foods and turning to novel foods.

### 4.3. Nanotechnologies

Nanoparticles incorporated into food products, food contact materials, or stable emulsions are expected to provide benefits such as stabilizing bioactive compounds; extending shelf-life, quality, and safety monitoring; and improving the sensory, textural, aroma, taste, consistency, and nutritional bioavailability of food [127,128]. The application of nanotechnologies can prolong the shelf-life of foods; prevent contamination; and increase food bioavailability, taste, texture, and consistency while producing safe and high-quality functional food [127], as well as the advantages of additional taste variants and health-promoting additives [24]. In recent years, the use of encapsulation and micro- and nanoencapsulation to develop new functional and fortified foods has gained momentum [83].

By using nanosensors and electronic tongue and nose signals, information can be provided about the characteristics of fruit odors, fruit aroma changes, quality determination in milk-like products, and monitoring of quality control processes [129], as well as information about toxins, contamination, and pesticides in foods [130]. The combination of nanoparticles such as silver, gold, zinc, iron, and copper with different medicinal aromatic plant essential oils and their components, such as carvacrol, p-cymene, thymol, and eugenol, can result in synergistic antimicrobial activity and will make important contributions to food preservation and shelf-life extension in the future. The bioavailability, efficiency, and stability of bioactive molecules such as vitamins, antioxidants, and food ingredients can be increased with nanoformulations.

Nanotechnology improves the taste, quality, and texture of food; is used for food quality, safety, nutrition, processing, packaging, and long-term storage; and can play an important role in designing higher-quality, sustainable, and healthier foods [127,128]. Additionally, public concerns regarding the use of such novel and unfamiliar technologies affect consumer acceptance. Therefore, the successful introduction of nanotechnological products into the food market is closely related to increasing knowledge, awareness, and trust, as well as the establishment of science-based regulation as a result of toxicology research [47]. Insufficient scientific knowledge regarding the potential risks of nanotechnology applications in terms of human health, safety, and the environment, as well as the lack of safety and environmental assessment, limits its spread [130].

### 4.4. Synthetic Biology

It is envisaged that synthetic biology technology, which is based on interdisciplinary integration for the targeted design, transformation, and even resynthesis of organisms and the design of living systems [131], will increase the production capacity of the food industry and reduce pollution and energy consumption by creating new species and technologies [132]. With synthetic biology technologies, the rapid production of food and food components such as proteins, lipids, and vitamins by microbes using environmentally friendly methods could be a promising alternative. The biological production of foods, considering nutrition, safety, quality, resource conversion efficiency, and the evaluation and product quality standards to be developed, will significantly contribute to the existing traditional food industry.

Although research in the field of food started late [133], synthetic biology technology has been used to design microbial food genomes and food ingredient synthesis pathways to ensure targeted, efficient, and accurate production of food ingredients, as well as to convert renewable raw materials into food ingredients, functional food additives, and nutritional chemicals [20]. Enzymatic hydrolysis and precision fermentation technology developed for the production of food ingredients not only enables the recovery of many valuable, bioactive, and functional components by utilizing byproducts but also helps sustainable food production and nutrition [83,134]. Advances in synthetic biology and fermentation technologies may enable the fabrication of protein and the cultivation of meat cells for food and feed [135,136]. The capabilities and mutually beneficial interactions of plants, fungi, and bacteria are critical for future food production and processing.

This system will contribute to food security, nutrition, and sustainable food supplies in the future, including the discovery of new food sources, the improvement of food nutrition, and the addition of new functions. In evaluating the sustainability of foods, the main framework is the life cycle assessment approach, and research on the environmental impact and changes in food consumption patterns and the development of innovative biobased products is a priority [137,138]. Functional healthy foods, new and high value-added food additives, precise nutrition and personalized food production, and artificial biosynthesis of food resources can be realized with synthetic biology techniques [20]. Precision nutrition and personalized food production tailored to each individual’s needs and differences in food sensory perception continue to be developing trends.

### 4.5. 3D Food Printing

Additive manufacturing, commonly known as 3D printing, is an emerging technology for creating customized and personalized food designs with complex geometric shapes, textures, and nutritional content. 3D food printing technology, which allows the customization of the shape, color, taste, and nutrition of food [139], can produce products layer by layer on the basis of a data model and from edible materials such as chocolate, dough, cheese, hydrogel, and meat [140], and has been applied to multiple food fields, such as military food and children’s and elderly foods and snacks [141]. In fact, meat products [142,143], bakery products and personalized nutrition [144], chicken meat-based products [145], and protein-rich snack foods [146] can be produced using 3D printing technology. 3D printing of food is an important method for achieving efficiency and energy savings; obtaining personalized, nutritious, and customized food [147]; overcoming the shortcomings of traditional food processing technology; improving environmental pollution and food shortages; and becoming an important component of the food industry [20].

With 3D food printing, as an emerging food processing technology, crispy, smooth, soft, and easy-to-chew foods can be produced for patients and elderly people who have difficulty chewing and swallowing, as well as snacks for children and young people [148]. 3D printing technology can be used to print dried food ingredients and achieve long shelf-life products, as well as personalized foods for different professions, genders, ages, and lifestyles [20]. In addition, 3D food printing technology is thought to aid in the preparation of customized food suitable for athletes and pregnant women [149]; reduce the waste of raw materials and energy caused by traditional manufacturing technologies [150]; provide personalized, high-productivity, and high-performance eco-foods with low environmental impact and cost [151]; contribute to the diversification of food styles and structures and the relative reduction of production costs [152]; modify the structure of food, creating soft foods and achieving better printability and fiber structure of new generation hybrid meat analogs [153]; and promote social development toward a more environmentally friendly and sustainable direction and play a greater role in future food manufacturing [20]. These new technologies, which expand from 3D printing to 4D, 5D, and 6D printing, along with software and smart material developments, are expected to lead to innovations in quality food production in the future [154].

### 4.6. Future Foods

Foods of the future are defined as foods that increase the level of production or offer the ability to reduce production costs and greenhouse gases while considering the environment, can be produced on a large scale under controlled conditions, are land efficient, and can develop rapidly as a result of technological developments [43]. In line with the goal of meeting human needs for a better life as part of sustainable development, the big food view has begun to attract attention [20]. To meet human needs, ensuring the supply of food in quantity, improving its function and quality, changing the traditional form of food supply, and diversifying and developing food resources in all directions and in multiple ways are necessary [155]. Although animal-derived foods such as meat are the best source of nutrient-rich foods [156], sustainable development goals and consumer awareness of health and environmental issues indicate that the demand for and consumption of vegan foods and plant-based diets will increase in the future [52]. While animal-based proteins have superior digestibility and bioavailability compared with plant-based proteins, alternative protein sources are considered more sustainable than animal proteins [157]. While rising incomes and falling food prices are increasing animal-based diets, global production and consumption of animal-based products, although remaining issues, will continue to increase in the future [158]. Meat production and consumption are often controversial and variable. Although reducing meat consumption is more advocated in developed countries, developing countries and poor people consume less meat and dairy products than rich countries.

In the future, the food industry will change the way food is produced through a high integration of food technology, biotechnology, and information technology, and in the future, food will be largely produced efficiently, environmentally friendly, and sustainable in industrial workshops [20]. In fact, if managed in accordance with human judgment, artificial intelligence in nanotechnology, microbiology, chemistry, agriculture, monitoring, and management can provide significant advances in food safety [159,160]. Advances in knowledge and technology and the future technological revolution can enable the production of nutritious and environmentally friendly food, increase and diversify production systems, and reduce environmental degradation, with improvements in resource use efficiency and profitability [135]. For the production of nutrients, future foods require less land than animal-sourced foods do, have less environmental impact, have an effective feed conversion ratio and a well-balanced amino acid composition, and could reduce competition for land and water for food, feed, fiber, and fuel production [43,99,161]. In the future, food production must find and overcome future challenges, especially water resource scarcity, temperature changes, food scarcity, and waste, in a cost-effective manner [160]. In the future, food systems must be able to deliver healthy, more environmentally sustainable, and risk-resistant diets. In the future, foods must be designed to meet the nutritional value of foods, the specific nutritional and health demands of different communities, and the quality of life [162].

With the development of biology and food technology, as food in the future expands toward richer biological resources, more agricultural products will move toward artificial synthesis and production. It is predicted that foods in the future may be more advantageous than traditional food industry products in terms of nutrition, health, safety, environmental protection, and cost [163]. In the future, based on innovative approaches that balance nutrition, health, sustainability, and environmental responsibility, foods can increase production levels and reduce production costs [7,161]. Feeding the growing world population limited in resources is becoming an increasingly major global challenge for the agri-food sector, and food choices are changing in ways that affect human health and the environment. One way to overcome these challenges is to improve soil nutrition on underperforming land and increase grain yield per unit area while reducing environmental impacts and food waste [161]. The other way is to make use of salty lands, develop and grow varieties that are resistant to abiotic stresses such as salinity and drought, domesticize resistant wild species, and evaluate the nutrition and eating quality of these salt-resistant varieties [164]. Another way to make future food systems more sustainable is to diversify agricultural systems, use local biodiversity, and research and utilize orphan crops and edible wild plants [165]. Some recommendations for sustainable food production and resilient food processing are given in Table 1. The impact of future foods on not only other environmental problems, such as water pollution, eutrophication, acidification, biodiversity, and air quality but also bioavailability, digestibility, allergies, and food safety, should be further investigated.

## 5. Possible Future Challenges and Limitations in Food Systems

Current and future challenges include emerging microorganisms and their toxins in food products; food allergens; co-optimization between safety and quality; improved nutrient availability for certain ages, characteristics, and lifestyles; alternative protein sources; processes that consume less water and energy; environmental, social, and economic concerns; and global warming. Climate change, greenhouse gas emissions, water–energy–food connections, and the need to protect limited natural resources are the most important obstacles to ensuring food security and a sustainable agriculture–food system [48]. These include issues such as inequitable food distribution, declining quality and nutritional adequacy, antimicrobial resistance, and food waste [135]. In addition, drought caused by global warming causes production losses may affect food safety and security, decrease yield and productivity, and increase irrigation costs [4], although the opposite may occur in some regions [39].

In sensitive regions, the strengthening tendency of farmers to quit farming after disasters reduces the population related to agriculture and local resource management and emerges as one of the important problems of the future [174]. Urban expansion, intensification, and peripheralization are observed in many parts of the world [175], and urban areas are being transformed into nonagricultural uses [176]. Uncontrolled urbanization leads to a decrease in cultivated agricultural areas, contrary to the principles of sustainability and food security [177]. The environmental, social, and climatic costs of agricultural production are increasing. Urbanization causes the transfer of the rural workforce to cities, and increases the demand for urban space and the need for food consumption, but it also causes a decrease in urban areas of arable land and food production. In the future, the combination of population and urbanization will put pressure on the local food supply, increase the nutritional burden in urban areas, and may change consumption patterns [125].

It is unclear whether the future will provide safe food and good nutrition due to climate change and its disproportionate impact on underdeveloped countries, water resource constraints, agri-food chain structures, limited resources, and increasing populations. Furthermore, research shows that decreasing the amount of land available for food production makes adequate access to food more difficult [178]; and affects food security due to the loss of biodiversity, climate change, and ecosystem services and increased competition for natural resources [171]; continues to increase the degradation and depletion of natural resources such as soil, forest, and water [179]; and increases food insecurity and health risks due to climate change disrupting agricultural production and food supplies [180].

The Mediterranean region is considered one of the regions most exposed to the effects of climate change, water scarcity, biodiversity loss, and land degradation. For these reasons, the main challenges and driving forces affecting the agricultural-food systems and water resources of the region are identified, and information and evidence-based recommendations are developed to take precautions and action against the challenges that the food sector faces [12]. In the Mediterranean region, which is a hotspot for climate change [181], there is an expectation of greater vulnerability to climate change and, in particular, increased frequency and severity of agricultural and hydrological droughts and greater pressure on the food and water sector, as well as reduced crop production and river flows [182].

While the increase in extreme weather events and climate change disrupt food supply chains, threaten sustainable crop production, exacerbate food insecurity, and make public health systems unhealthy [183,184,185], making production regions sensitive to external shocks and limiting their contribution to a reliable food system [186], the weakness of economic systems and dependence on exports and imports of basic products cause malnutrition [21]. Although access to food is a primary need, it is not equal everywhere on the planet, and as populations grow, not everyone can be guaranteed access to healthy and nutritious food [24]. Because the challenges in agri-food systems are complex, wide-ranging, and closely interconnected, comprehensive studies of regional food systems, from agricultural impacts on resources to food waste, are still very limited [12]. Even in the best countries in terms of food safety, it is important to adapt and review practices constantly, as risks can change over time [159]. The main challenge facing agriculture today is not only ensuring food security and sustainability while improving the environment without stagnating agricultural productivity but also competing in the globalizing market and adapting to changing consumer demand and eating habits.

In the context of food security, the neglect of risk management, the combination of vulnerability and uncertainty surrounding the development of the system for the future, contributes to a growing scarcity of food. In this context, developing and promoting sustainable solutions to reduce risks and increase food security [2], as well as healthy management of online food safety governance, is inevitable and has become a new trend [187]. Grains and grain-based products, which constitute the basic food for a large part of the world’s population, can be contaminated by microorganisms and mycotoxins during harvest, transportation, distribution, and especially long-term storage, causing economic losses and health problems [188,189]. Since mycotoxins cause a loss of almost one-third of grain production every year and make food unsuitable for consumption, new technologies should be developed to reduce the postharvest loss of grains to feed the growing world population with limited resources and relieve pressure on the agri-food sector. Emerging technologies using nonthermal or optimized thermal processing, such as cold plasma technology (CPT), ultrasound, high pressure, pulsed electric field, pulsed light technology, and microwave processing, have significant potential to improve the properties of native starch and meet the demand for minimal processing, preserving bioactive compounds and aiming to ensure food safety, affordable food products with better organoleptic and nutritional properties [109,190]. Among the new techniques developed for the degradation of mycotoxins in the food industry, such as CPT, irradiation, biological methods, and ozone treatment, CPT is especially suitable for surface decontamination of cereals and grains [188].

Because of climate change and the need for toxic and chemical treatment in food and agriculture, CPT has emerged as a low-cost, environmentally friendly, effective, sustainable, and nonthermal technology that ensures food safety [191,192]. CPT has gained considerable attention as a promising approach for reducing postharvest losses and controlling fungi and mycotoxins in grains and crops, as well as ensuring food safety through sustainable practices [193]. This technology has the potential to inactivate enzymes and increase the antioxidant level of food products, preventing microbial contamination while preserving the nutritional and sensory qualities of foods and the properties of plant-based proteins [192]. CPT, which does not require chemicals, does not leave toxic byproducts, and does not adversely affect the nutritional and sensory properties of food, is used to reduce the microbial count, breakdown mycotoxins, inactivate enzymes, and reduce pesticides and allergens in food products [191,194]. Research has shown that CPT can inactivate pathogenic microorganisms on the surface of cereal grains, improve the microbial safety of products, ensure consumer health and safety, extend shelf-life, and improve the properties of grain starch [189,195]. CPT, a promising alternative to heat treatment techniques, can ensure food quality and safety. Owing to the challenges of the food industry arising from the risk of mycotoxin contamination, there is an imperative need to develop and implement commercial-scale sustainable mycotoxin-degrading technologies [196].

Sustainable food production and reducing the environmental impact of production require the use of food industry byproducts not as waste but as a source of bioactive compounds and raw materials for food production [197]. Solutions are needed to address the challenges of food and nutrition insecurity, replace animal-based protein sources, and meet the demand for convenient, nutritious, and health-promoting foods, as well as functional ingredients and biologically active and pharmaceutically important phytochemicals [198,199,200]. Owing to the demand for safe and processed meats, optimization of processing conditions and innovative technologies such as cold plasma, microwaves, irradiation, high-pressure thermal processing, and multitarget preservation are needed to reduce chemical preservatives, preserve the sensory and nutritional properties of processed meats and ensure their microbial safety [201]. In fact, consuming fruits and vegetables, even underutilized but promising and stress-resistant vegetables rich in bioactive compounds, phytochemicals, and antioxidants, is an important solution for balanced mineral and vitamin intake in addition to potential health benefits [178]. The two main future research trends, increasing food production and reducing food waste to ensure food security [48], will be affected by challenges such as climate change, population growth, population aging, inadequate supply of safe food, coordination of the relationship between food security and the ecological environment, and increasing food demand [30]. Although other food trends emerge with physical, biological, and digital technological developments, the sensory and nutritional properties of foods can be developed and improved, as well as contributing to their sustainability.

## 6. Conclusions

Food–energy–water–agricultural systems need to be built and developed. Although challenging, sustainable innovations for food sustainability and technologies that use renewable energy and have less environmental impact are urgently needed. Cultivation patterns adapted to climatic conditions, effective irrigation programs according to plants and regions, and plant varieties tolerant to changing temperatures should be developed and encouraged, and even the planet’s resources should be protected from humans.

With the widespread use of certificates and labels to build trust between consumers and producers, the establishment of a science-based regulatory framework, and the increase in public knowledge, awareness, and trust, innovations in the food industry and consumers’ attitudes toward new and functional foods must be investigated. Investigating trends in future alternative protein sources and the effects of processing is critical. In the future, the sustainable use of all raw materials, byproducts, and side streams, and the development of more new products and protein sources are essential for food safety and human health. In the development of new products and protein sources, it is necessary to ensure safe conditions, including microbial safety, chemical contaminant and allergenicity concerns, and consumer concerns, by filling legal gaps. The food industry needs to direct consumption toward sustainably produced foods and develop strategies that can reduce food waste by focusing on the protection of the environment and natural resources. To ensure food safety, security, and sustainability, food production should increase, its effects on the environment should be evaluated, and innovative research, data, techniques, and perspectives should be developed. Nutritional and functional properties can be improved by improving protein quality, digestibility, and bioavailability by blending different protein types, developing fortification techniques, and optimizing processing conditions.

In the future, traditional and modern foods based on artificial intelligence, synthetic biology, additive manufacturing, and other new technologies reflecting production methods and lifestyles will be developed. Modern science and technological innovations will certainly transform the food supply, but before implementation, it is essential to collaborate across different scientific disciplines and evaluate and reduce the risks of new technologies.

## Figures and Tables

**Table 1:** Some priority strategies, recommendations, and actions for improving the resilience of future food systems and sustainable food production.

Strategies/Recommendations/Actions	Ref.
Focusing on integrated agricultural reform and agriculture	[16]
Technological change Adoption of sustainable production systems and practices Make food systems more efficient, inclusive, and resilient Protecting biodiversity, and ensuring ecosystem services	[20]
Promote agroecology and agroecological techniques Biofortification and sustainable intensification	[21]
Diversified farming and production systems Creating sustainable and resilient farming and production systems Regenerative and mixed agricultural practices The retention and restoration of natural ecosystems	[22]
Climate-smart agriculture Resource use efficiency	[33]
Transitioning to a circular and resource-efficient economy	[34]
Reducing waste and pollution from agricultural contaminants	[36]
Increasing the photosynthetic optimum in cultivated plants	[39]
Promote of sustainably sourced plant proteins as promising strategy	[49]
Research, development and evaluation of alternative protein sources	[51]
Promote technological improvements in meat production Change in meat consumption	[90]
Sustainable irrigation expansion and agricultural intensification Ensure sustainable consumption patterns by moderating diets and reducing food losses	[112]
Improve microbial protein production	[121]
Develop precision fermentation for food ingredients	[134]
Creating technological innovation for the transition to circular agriculture Identifying technologies with co-benefits Developing the capacity to engage in technological advances	[135]
Diversify agricultural systems, use local biodiversity, orphan crops, and wild edible plants	[165]
Redistribute cropland, improve water-nutrient management, reduce food waste, and change diets	[166]
Develop artificial photosynthesis systems	[167]
Developing local community-based urban agriculture	[168]
Combine sustainable agriculture with flexible food processing and sustainable consumption	[169]
Transforming bio-waste into value-added products such as bio-based fertilizer	[170]
Promote resource and biodiversity conservation Develop agroforestry and tree-based farming Diversify crops and use climate resilient cultivars and neglected and under-utilized plants Divert towards plant food and food biofortification	[171]
Increasing soil, water, energy, fertilizer production efficiency Recycling waste and upcycling byproducts Development new energy generation systems Implementation of reduced energy use systems Discover new food sources Increasing biodiversity, protecting genetic diversity, ending the loss of ecosystems, and expanding their restoration	[172]
Increasing climate smart agriculture Improving livestock, sustainable fisheries, and grazing management Reducing pressure on ecosystems and food waste	[173]

## Data Availability

No new data were created or analyzed in this review. Data sharing is not applicable to this article.
